# Looking Beyond Dose: Identifying Responders and Non-Responders to RehaCom Computerized Cognitive Rehabilitation in Progressive MS—The CogEx Study

**DOI:** 10.1177/15459683261432556

**Published:** 2026-04-25

**Authors:** Cecilia Meza, Roberto S. Hernandez, Silvana L. Costa, Amber Salter, Nancy D. Chiaravalloti, Maria Pia Amato, Giampaolo Brichetto, Jeremy Chataway, Gary Cutter, Ulrik Dalgas, John DeLuca, Rachel Farrell, Peter Feys, Massimo Filippi, Jennifer Freeman, Matilde Inglese, Robert W. Motl, Maria A. Rocca, Brian M. Sandroff, Anthony Feinstein, CogEx Research Team

**Affiliations:** 1 Department of Psychiatry, Sunnybrook Health Sciences Centre, Toronto, ON, Canada; 2 Division of Neurology, St. Michael’s Hospital, Toronto, ON, Canada; 3 Department of Neurology, Section on Statistical Planning and Analysis, Dallas, TX, USA; 4 Kessler Foundation, Neuropsychology and Neuroscience Center, East Hanover, NJ, USA; 5 Department of Physical Medicine and Rehabilitation, Rutgers New Jersey Medical School, Newark, USA; 6 NEUROFARBA Department, Section of Neurosciences, University of Florence, Italy; 7 Scientific Research Area, Italian Multiple Sclerosis Foundation (FISM), Genoa, Italy; 8 Queen Square Multiple Sclerosis Centre, Department of Neuroinflammation, UCL Queen Square Institute of Neurology, Faculty of Brain Sciences, University College London, UK; 9 National Institute for Health and Care Research, University College London Hospitals Biomedical Research Centre, UK; 10 Department of Biostatistics, University of Alabama at Birmingham, USA; 11 Department of Public Health, Exercise Biology, Aarhus University, Denmark; 12 REVAL Rehabilitation Research Center, Faculty of Rehabilitation Sciences, Hasselt University, Diepenbeek, Belgium; 13 Neuroimaging Research Unit, Division of Neuroscience, IRCCS San Raffaele Scientific Institute, Milan, Italy; 14 Neurology Unit, IRCCS San Raffaele Scientific Institute, Milan, Italy; 15 Vita-Salute San Raffaele University, Milan, Italy; 16 Faculty of Health, School of Health Professions, University of Plymouth, Devon, UK; 17 Department of Neuroscience, Rehabilitation, Ophthalmology, Genetics, Maternal and Child Health, and Center of Excellence for Biomedical Research, University of Genoa, Italy; 18 Department of Kinesiology and Nutrition, University of Illinois Chicago, USA; 19 Temerty Faculty of Medicine, Department of Psychiatry, University of Toronto, ON, Canada

**Keywords:** progressive multiple sclerosis, computerized cognitive rehabilitation, processing speed, symbol digit modalities test (SDMT), cognitive impairment

## Abstract

**Background:**

RehaCom, a computerized cognitive rehabilitation program for people with multiple sclerosis (PwMS), has been studied primarily in relation to treatment “dose” (duration, frequency, and adherence), with less focus on which training modules drive improvement or which participant factors predict responsiveness. This secondary analysis of the CogEx trial investigated whether progression within specific RehaCom modules was associated with processing speed improvement, measured by the SDMT, and whether baseline characteristics predicted response in participants with progressive MS.

**Methods:**

A total of 153 participants completed 12 weeks of RehaCom training across 5 attention-based modules. Cognition was assessed at baseline, 12 weeks, and 6 months using the SDMT. Correlation and regression analyses evaluated associations between module progression and cognitive outcomes.

**Results:**

Progression correlated significantly with SDMT improvement in 4 modules, with the strongest effects for Attention/Concentration (*r* = .37, *P* < .001) and Divided Attention-2 (*r* = .36, *P* < .001). Higher baseline SDMT, higher premorbid IQ, older age, and greater module progression independently predicted better SDMT performance at12-weeks (adjusted *R*^2^ = .73). At 6 months, higher baseline SDMT, greater progression in Attention/Concentration and Divided Attention-2, older age, and female sex predicted better SDMT performance (adjusted *R*^2^ = .71).

**Conclusion:**

Processing speed gains in progressive MS were related to both module-specific progression and participant characteristics, supporting a precision approach to cognitive rehabilitation that tailors training content to individual cognitive profiles. This study is a secondary analysis of the CogEx trial (ClinicalTrials.gov Identifier: NCT03679468; https://clinicaltrials.gov/ct2/show/NCT03679468).

## Introduction

Computerized cognitive rehabilitation (CCR) has emerged as a promising intervention for improving cognition in people with multiple sclerosis (PwMS).^1^ Among available programs, RehaCom is the most widely studied.^2^ However, prior research has emphasized treatment “dose” (frequency, duration, and adherence), with less focus on the effectiveness of specific modules or participant characteristics that distinguish responders from non-responders—factors essential for optimizing rehabilitation.

Evidence linking participant profiles to CCR outcomes remains limited. Only 1 study—Sandroff et al,^3^ a secondary analysis of the CogEx trial—specifically examined RehaCom. Intact learning and memory at baseline, together with higher premorbid IQ (a proxy for cognitive reserve), predicted greater improvements in processing speed (SDMT). Other work using non-RehaCom interventions has reported mixed findings. Taylor et al^4^ identified younger age, relapsing–remitting MS, absence of recent relapses, stronger psychological well-being, and better baseline memory and processing speed as predictors of improvement (CRAMMS intervention). In contrast, Prouskas et al^5^ reported that greater baseline impairment, particularly in attention and processing speed, predicted the largest gains with C-Car computerized attention training. Neuroimaging results, however, aligned more consistently with prior work, showing that better preserved brain structure—larger brain volume and lower T2 lesion burden—was associated with responsiveness, highlighting the value of early intervention.

These inconsistencies underscore a critical gap: it remains unclear whether specific RehaCom modules, and the extent of engagement with them (eg, level completion and progression), are most effective, and how individual variability shapes outcomes. The present exploratory study addressed this gap by examining the influence of RehaCom modules and participant characteristics in progressive MS, using a subset of CogEx participants—a multicenter, 4-arm randomized trial of CCR and aerobic exercise across 11 sites in 6 countries.^6^

## Method

### Participants

This study included 153 participants, a subset of 311 PwMS enrolled in the CogEx trial, reported previously.^6,7^ The initial RehaCom group comprised 155 participants; however, 2 were excluded due to outlier SDMT change scores (>±10 points over 12 weeks). This threshold has been applied in prior MS cognitive rehabilitation research to identify changes unlikely to represent true treatment effects and more likely attributable to measurement error or nonspecific factors.^8,9^

This subset comprised all participants randomized to the computerized cognitive rehabilitation (RehaCom) intervention who completed the 12-week training protocol and had complete cognitive outcome data. Baseline demographic and clinical characteristics of this subset were comparable to those of the full CogEx cohort, as reported in the parent trial.

Eligible participants had a confirmed diagnosis of primary or secondary progressive MS, were 25 to 65 years of age, and demonstrated cognitive impairment, defined as SDMT performance ≥1.282 SD below normative data (10th percentile). Exclusion criteria included EDSS ≥7.0, a history of other central nervous system disorders, and corticosteroid use or MS relapse within 3 months. The exclusion criterion of “other central nervous system disorders” referred to neurological conditions; a prior diagnosis of attention-deficit/hyperactivity disorder (ADHD) was not an explicit exclusion criterion. All participants provided written informed consent.

### Study Design

Participants in this analysis were those who received the RehaCom computerized cognitive rehabilitation intervention (Hasomed Inc., Magdeburg, Germany; http://www.hasomed.de), comprising 49% of the total CogEx sample, irrespective of whether it was combined with aerobic (EX) or sham exercise (EX-S). Given that the primary outcome measure in CogEx was processing speed as measured by the SDMT, cognitive rehabilitation targeted this domain using 5 attention-based modules: Divided Attention 1, Divided Attention 2, Attention/Concentration, Vigilance 2, and Sustained Attention.

Training was delivered twice weekly over 12 weeks (24 sessions total), with each participant completing 48 module administrations (2 modules per session). Training parameters were set at 20 minutes per module, for a total of 40 minutes per session. The 48 module administrations were distributed across the 5 RehaCom modules as follows: Sustained Attention, Divided Attention 1, and Divided Attention 2 were each administered 10 times, while Attention/Concentration and Vigilance 2 were each administered 9 times, yielding 48 module administrations.

### Procedure

Participants began each module at level 1, following a standardized sequence (Supplemental Figure 1). The fixed order of RehaCom modules and uniform starting levels were selected to ensure standardization across the multi-site, multi-country CogEx trial. Although participants differed in baseline cognitive functioning, task difficulty within each module was auto-adaptive, adjusting in real time based on individual performance to support engagement and mitigate boredom or frustration. Progression to subsequent levels within each RehaCom module was determined solely and automatically by the software’s built-in adaptive algorithm, based on participants’ real-time performance. Advancement decisions were fully automated and were not influenced by therapist judgment or manual adjustment. Progression was supervised by a research assistant, with higher levels introducing greater stimulus complexity and distractors.

Module descriptions and maximum levels were:

■ Divided Attention 1: train conductor task; monitor controls and railway signs, adjust speed, and resist distractions (max = 14).
■ Divided Attention 2: driving task; monitor dashboard/roadway, respond to cues, and ignore distractors (max = 22).
■ Attention/Concentration: visual search task; locate a target image within a distractor matrix, requiring sustained focus (max = 24).
■ Vigilance 2: factory inspector task; detect mismatched items on a conveyor belt under low stimulus frequency (max = 9).
■ Sustained Attention: quality-control task; detect frequent deviations on a fast-moving conveyor belt under time pressure (max = 9).

Given variability in the total number of difficulty levels across RehaCom modules, progression was quantified using 2 indices:

1. Final-level completion (Index 1): the percentage indicating whether a participant completed the module in full, reflecting training *breadth*.
2. Maximum difficulty attained (Index 2): calculated as *(highest level* *÷* *total levels)* *×* *100*, reflecting training *depth*.

Index 1 was used for descriptive purposes, whereas Index 2 served as the primary predictor in statistical analyses. Because RehaCom modules differ substantially in their total number of difficulty levels (ranging from 9 to 24), absolute level counts are not directly comparable across modules; accordingly, progression was operationalized as the percentage of total levels completed (Index 2), thereby standardizing progression relative to the maximum difficulty available within each module.

Importantly, the degree of difficulty change between successive levels is determined by RehaCom’s proprietary adaptive algorithm and cannot be independently quantified. Although advancing levels generally reflect increased stimulus complexity and the introduction of distractors, the magnitude of difficulty change between levels is not assumed to be equivalent across modules, nor is it assumed that modules with fewer levels necessarily involve larger difficulty increments per level. Accordingly, Index 2 captures relative progression within a given module rather than absolute or directly comparable task difficulty across modules. This consideration was taken into account when interpreting module-specific associations with cognitive outcomes.

### Measures

Cognitive assessments were conducted at baseline, 12 weeks, and 6 months using alternate versions of the Brief International Cognitive Assessment for MS (BICAMS).^10^ The battery includes the Symbol Digit Modalities Test (SDMT), a measure of information processing speed, which served as the primary outcome. Verbal learning and memory were assessed using the California Verbal Learning Test-Second Edition (CVLT-II), while visuospatial learning and memory were assessed using the Brief Visuospatial Memory Test-Revised (BVMT-R). Country-specific normative data with corrections for age, sex, and education were applied.^6^

Adherence was tracked with Weekly Intervention Adherence Forms that documented attendance, reasons for missed sessions, modules completed, maximum levels attained, session duration, and qualitative comments. In this secondary study, all participants completed the intervention. The parent study reported high adherence, facilitated by taxi transportation to appointments and a 2-week window for makeup sessions.^6^

## Statistical Analysis

Baseline characteristics (age, education, disease duration, EDSS, and baseline cognitive scores) were summarized using descriptive statistics. Continuous variables were assessed for normality using visual inspection of histograms and the Shapiro–Wilk test. Normally distributed variables are reported as mean ± SD and compared between groups using 2-sample *t*-tests, whereas non-normally distributed variables are reported as median [IQR] and compared using Wilcoxon rank-sum tests. Categorical variables are presented as counts and percentages and compared using Pearson’s chi-squared tests.

All participants received RehaCom training. Before pooling participants for the main analyses, we verified that module completion rates (Index 1) and demographic or cognitive characteristics did not differ between the 2 subgroups: cognitive rehabilitation plus aerobic exercise (EX) and cognitive rehabilitation plus sham exercise (EX-S). Participants were first dichotomized according to whether they reached the final level of each module (Index 1 = 100%), and cognitive outcomes were compared between these subgroups. As no significant baseline differences were observed between the EX and EX-S groups, and module completion rates were comparable, participants from both arms were combined for all subsequent analyses.

Associations between module progression and change in SDMT were examined using Pearson correlations, with the maximum difficulty level attained (Index 2, expressed as a percentage of the total possible) as the predictor. Although some module distributions were skewed and displayed ceiling effects, SDMT change scores were approximately normally distributed; therefore, Pearson’s *r* was used to quantify linear associations.

For bivariate analyses, SDMT change scores were calculated as the difference between follow-up and baseline performance (ie, SDMT at 12 weeks minus baseline SDMT). In contrast, multivariable regression analyses used raw SDMT scores at 12 weeks and at 6 months as the outcome variables, with baseline SDMT included as a covariate to account for individual differences in initial performance.

Multivariable linear regression analyses were conducted to identify predictors of 12-week SDMT performance. Candidate predictors included demographic variables (age and education), disease-related measures (disease duration and EDSS), baseline cognitive scores (SDMT, CVLT-II, and BVMT-R), and RehaCom progression indices (maximum level attained and final-level completion). Given the relatively large number of candidate predictors compared with the sample size, stepwise regression with AICc-based variable selection was employed to balance parsimony and explanatory power while minimizing overfitting.

Candidate predictors for all regression models were defined a priori and included demographic variables (age, sex, and education), disease-related measures (disease duration and EDSS), baseline cognitive performance (SDMT, CVLT-II, BVMT-R, and premorbid IQ), and RehaCom progression indices. The same pool of candidate predictors was entered into stepwise regression models at each time point. Variables were retained based on AICc-based model selection, reflecting their independent contributions after accounting for collinearity and shared variance. Consequently, predictors retained at 12 weeks and 6 months differed slightly, reflecting timepoint-specific effects rather than differences in model specification.

Two regression strategies were applied: 1 incorporating individual RehaCom module progression variables and another using a composite attention measure (Attention/Concentration, Divided Attention-1, and Divided Attention-2).

The same analyses were performed for 6-month SDMT change and for the secondary cognitive outcomes (CVLT-II and BVMT-R). A composite variable was created by averaging the percentage of total levels completed across the Attention/Concentration, Divided Attention-1, and Divided Attention-2 modules, which showed strong intercorrelations and greater variability across participants. Vigilance-2 and Sustained Attention were excluded from the composite and regression analyses because most participants reached the final level (71% and 84%, respectively), resulting in ceiling effects that limited variability. Statistical significance was set at *P* < .05. All analyses were performed using SAS version 9.4 (SAS Institute, Cary, NC, USA).

For descriptive purposes, we also calculated the proportion of participants demonstrating clinically meaningful SDMT improvement, defined as an increase of ≥8 points.

## Results

### Participant Characteristics

The analysis included 153 participants who completed RehaCom. Overall, the cohort was middle-aged (median age: 53 years), 61% female, with a median of 13 years of education and a median disease duration of 14 years. Median EDSS score was 6, and baseline cognitive performance averaged 33 (SD = 8) on the SDMT, 45 (SD = 12) on the CVLT-II, and 21 (SD = 7) on the BVMT-R. Participants were equally distributed between the EX group (n = 75) and the EX-S group (n = 78). No significant differences in demographic, clinical, or baseline cognitive characteristics were observed between groups (Table 1).

**Table 1.** Baseline Characteristics of the Study Sample.

Characteristic	Total (N = 153)	CCR + EX (n = 75)	CCR + EX-S (n = 78)	*P*-value*
Age, years, median [IQR]	53 [48, 59]	53 [48, 58]	53 [48, 59]	.9
Female, n (%)	94 (61)	49 (65)	45 (58)	.3
Years of education, median [IQR]	13 [12, 16]	13 [11.5, 16.5]	13 [12.3, 16]	.7
Disease duration, years, median [IQR]	14 [7, 21]	14 [7, 22]	14 [7, 20]	.9
EDSS score, median [IQR]	6 [4.5, 6.5]	6 [4.5, 6.5]	6 [4.5, 6.4]	.7
Baseline SDMT, mean ± SD	33 (8)	32 (9)	33 (7)	.6
Baseline CVLT-II, mean ± SD	45 (12)	45 (12)	44 (11)	.8
Baseline BVMT-R, mean ± SD	21 (7)	21 (7)	21 (7)	.5

Abbreviations: SDMT, Symbol Digit Modalities Test (information processing speed); CVLT-II, California Verbal Learning Test-Second Edition (verbal learning and memory); BVMT-R, Brief Visuospatial Memory Test-Revised (visuospatial learning and memory).

Values are reported as mean (SD), median [interquartile range, IQR], or n (%), as appropriate.

Total = Combined CCR regardless of assigned exercise group.

CCR + EX = computerized cognitive rehabilitation (RehaCom) combined with aerobic exercise.

CCR + EX-S = computerized cognitive rehabilitation (RehaCom) combined with sham exercise.

* *P*-values are based on 2-sample t-tests, Wilcoxon rank-sum tests, or Chi-squared tests.

### Module Completion Rates

Completion was defined as reaching the final level of a given module (Index 1 = 100%). In the total sample (N = 153), completion rates varied by module: Sustained Attention (84%), Vigilance-2 (71%), Attention/Concentration (23%), and 0% for both Divided Attention-1 and Divided Attention-2 (Table 2).

**Table 2.** Final-level Completion Rates (Index 1) by RehaCom Module and Exercise Group Assignment.

Module	Highest level in module	Total N = 153 (%)	EX N = 75 (%)	EX-S N = 78 (%)	*P*-value^a^
Attention/concentration	24	35 (23)	18 (24)	17 (22)	.7
Vigilance 2	9	108 (71)	50 (67)	58 (74)	.3
Sustained attention	9	129 (84)	62 (83)	67 (86)	.6
Divided attention 1	14	0 (0)	0 (0)	0 (0)	1.0
Divided attention 2	22	0 (0)	0 (0)	0 (0)	1.0

Index 1 = final-level completion. Values represent the proportion of participants who reached the final level of each module.

CCR+EX = computerized cognitive rehabilitation (RehaCom) combined with aerobic exercise.

CCR+EX-S = computerized cognitive rehabilitation (RehaCom) combined with sham exercise.

Total = Combined CCR regardless of assigned exercise group.

a Pearson’s chi-squared test.

### Correlations with SDMT Change

**Figure 1.**
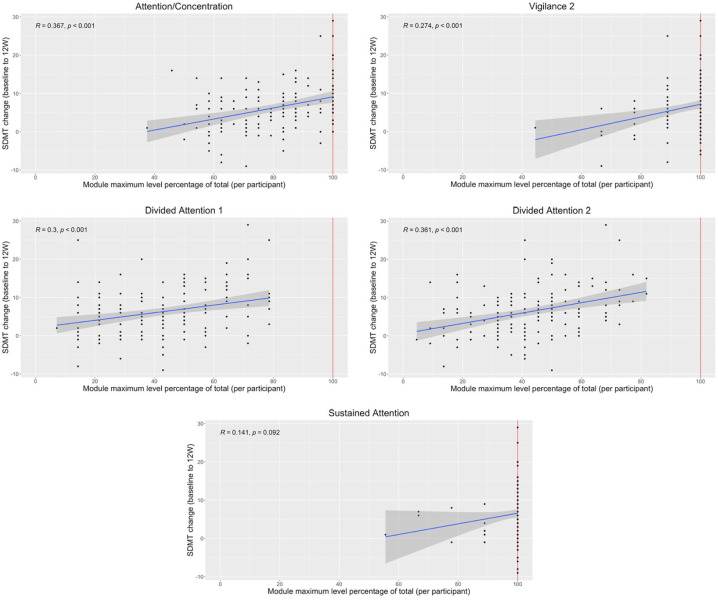
Associations between SDMT change (baseline to 12 weeks) and RehaCom progression by Index 2 (maximum difficulty attained, %) across individual modules. Index 2 = maximum difficulty attained, expressed as (highest level attained ÷ total module levels) × 100. The red line marks the maximum attainable level for each module. Significant moderate positive correlations were observed in 4 of the 5 modules (all *P* < .05), with the exception of Sustained Attention (*P* = .055). Distributions for Vigilance 2 and Sustained Attention were notably skewed, reflecting ceiling effects.

**Figure 2.**
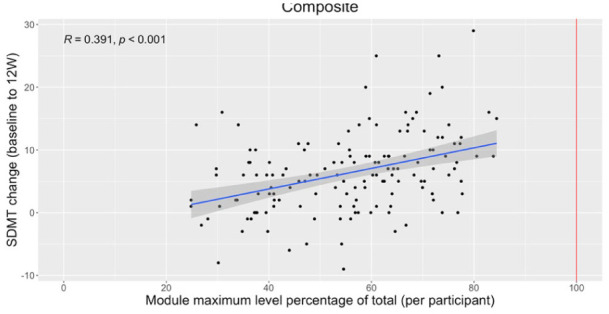
Association between SDMT change (baseline to 12 weeks) and composite attention progression (Index 2). Composite Index 2 = average percentage of maximum difficulty attained across the Attention/Concentration, Divided Attention-1, and Divided Attention-2 modules.

Progression, measured as the percentage of the maximum difficulty level achieved (Index 2), was significantly correlated with SDMT improvement at 12 weeks in 4 of the 5 modules (all *P* < .05), with the exception of Sustained Attention (*P* = .092). The strongest associations were observed for Attention/Concentration (*r* = .367, *P* < .001), Divided Attention-2 (*r* = .361, *P* < .001), and the composite measure combining Attention/Concentration, Divided Attention-1, and Divided Attention-2 (*r* = .391, *P* < .001; Table 3, Figures 1 and 2). Divided Attention-1 also showed a significant correlation with SDMT change (*r* = .300, *P* < .001), but no participants reached its final level, limiting further analysis of completion effects.

**Table 3.** Correlations Between RehaCom Progression by Index 2 (Maximum Difficulty Attained, %) and SDMT Change (Baseline to 12 weeks).

Variable	Correlation with SDMT change (baseline—12 weeks) (*r*)	*P*-value
Age	.138	.099
Gender (ref: male)^a^	−.013	.872
Total years of school	.064	.447
Duration of MS	.071	.398
Premorbid IQ	.317	<.001
Baseline SDMT	.126	.134
Baseline CVLT-II	.013	.876
Baseline BVMT-R	.293	<.001
RehaCom modules (percentage of maximum difficulty level completed)^b^		
Attention/Concentration	.367	<.001
Divided Attention 1	.300	<.001
Divided Attention 2	.361	<.001
Sustained Attention	.141	.092
Vigilance 2	.274	.001
Composite including Attention/Concentration, Divided Attention 1, Divided Attention 2	.391	<.001

Abbreviations: SDMT, Symbol Digit Modalities Test (information processing speed); CVLT-II, California Verbal Learning Test-Second Edition (verbal learning and memory); BVMT-R, Brief Visuospatial Memory Test-Revised (visuospatial learning and memory).

SDMT change was calculated as SDMT score at 12 weeks minus baseline SDMT score.

Gender correlations are ^a^ point-biserial correlation.

b Index 2 = maximum difficulty level attained, expressed as (highest level attained ÷ total module levels) × 100. Pearson correlations were used.

Using the percentage of total levels completed (Index 1), participants who reached the maximum level of Attention/Concentration demonstrated greater SDMT gains than those who did not (mean change = 9.9 vs 5.2; *P* < .001). Comparable effects were observed for Vigilance-2 (7.1 vs 4.0; *P* = .012) and Sustained Attention (6.7 vs 2.8; *P* < .001). Because most participants reached the final level in these 2 modules, ceiling effects limited their usefulness in regression and composite models. No significant effects were observed for CVLT-II or BVMT-R (Table 4).

**Table 4.** Cognitive Outcome Changes (Baseline to 12 weeks; mean ± SD) by Index 1 (Final-level Completion Status).

Module	Final level reached (%)^a^	SDMT change (mean ± SD)	*P*-value	CVLT-II change (mean ± SD)	*P*-value	BVMT-R change (mean ± SD)	*P*-value
Attention/concentration	Yes (23)	9.9 ± [6.8]	<.001	1 ± [7]	.3	−0.6 ± [4.8]	.4
	No (77)	5.2 ± [5.7]		2 ± [8]		−1.5 ± [6.0]	
Vigilance 2	Yes (71)	7.1 ± [6.2]	.012	2 ± [7]	.092	−1.2 ± [6.0]	.9
	No (29)	4.0 ± [6.1]		0 ± [7]		−1.3 ± [5.2]	
Sustained attention	Yes (84)	6.7 ± [6.5]	<.001	2 ± [7]	.11	−1.1 ± [5.8]	.2
	No (16)	2.8 ± [3.3]		−2 ± [9]		−2.9 ± [5.5]	

Abbreviations: SDMT, Symbol Digit Modalities Test (information processing speed); CVLT-II, California Verbal Learning Test-Second Edition (verbal learning and memory); BVMT-R, Brief Visuospatial Memory Test-Revised (visuospatial learning and memory).

No participants reached the final level in Divided Attention-1 or Divided Attention-2.

a Index 1 = final-level completion, coded as yes (100%) vs. no (<100%). Group comparisons were performed using 2-sample t-tests.

### Clinical Meaningfulness of SDMT Improvement

To contextualize the clinical relevance of observed SDMT changes, we examined the proportion of participants demonstrating clinically meaningful improvement, defined as an increase of ≥8 points on the SDMT at 12 weeks, consistent with prior CogEx analyses. Using this threshold, approximately 40% of participants met criteria for clinically meaningful improvement. This proportion is comparable to that reported in the parent CogEx trial, where 37% of participants demonstrated meaningful SDMT gains (see Supplemental Table 3).

### Multivariable Regression at 12 Weeks

Stepwise regression identified premorbid IQ, baseline SDMT, age, and maximum difficulty levels achieved (Index 2) in Attention/Concentration and Divided Attention-2 as independent predictors of 12-week SDMT performance (Table 5). Higher values on each predictor were associated with better outcomes, with baseline SDMT contributing most strongly (β = .72, *P* < .001). Attention/Concentration progression was the most robust intervention-specific predictor. The model explained 73.2% of the variance in 12-week SDMT scores (adjusted *R*^2^ = .732).

**Table 5.** Multivariable Regression Predicting 12-Week SDMT Scores Using RehaCom Progression Variables.

Predictor variable	Model 1: individual modules (Index 2)			Model 2: composite attention measure (Index 2)		
	β	95% CI	*P*-value	β	95% CI	*P*-value
Premorbid IQ	.13	0.01-0.24	.027	.13	0.02-0.25	.021
Baseline SDMT score	.72	0.53-0.92	<.001	.79	0.61-0.96	<.001
Max Level %—Attention/Concentration	.17	0.06-0.28	.004	—	—	—
Max Level %—Divided Attention 2	.09	0.01-0.18	.035	—	—	—
Composite (Attention/Concentration, Divided Attention 1 and 2)	—	—	—	.23	0.14-0.32	<.001
Age^a^	.15	0.01-0.29	.039	.14	0.00-0.28	.055
Adjusted *R*^2^	.732			.728		

Index 2 = maximum difficulty attained, expressed as a percentage of each module’s maximum. Stepwise linear regression with AICc-based selection was applied.

Although age was not significantly correlated with SDMT change in bivariate analyses, it entered the multivariable model because it explained unique variance in 12-week SDMT scores after adjustment for other predictors. *Divided Attention-1*, despite being significantly correlated with SDMT change, was excluded from the individual-module model due to collinearity with *Attention/Concentration* and *Divided Attention-2*, which accounted for stronger independent effects.

All predictors shown were selected from a common a priori—defined candidate pool using AICc-based stepwise regression.

a Age is retained in both models for transparency, although it was not an independent predictor once the composite variable was included.

When the individual module variables were replaced with the composite measure of Attention/Concentration, Divided Attention-1, and Divided Attention-2, results were comparable (β = .23, *P* < .001; adjusted *R*^2^ = .728; Table 5).

### Exploratory 6-Month Analyses

At 6 months, baseline SDMT remained the strongest predictor of SDMT outcomes (β = .69, *P* < .001; Supplemental Table 1). Module progression measured as maximum difficulty level achieved (Index 2) in Attention/Concentration (β = .21, *P* < .001) and Divided Attention-2 (β = .15, *P* = .005) also predicted performance. In addition, female sex (β = 2.6, *P* = .034) and older age (β = .22, *P* = .006) were associated with higher SDMT scores. The final model explained 70.5% of the variance in SDMT at 6 months (adjusted *R*^2^ = .705). A parallel model using the composite attention measure produced similar results (β = .32, *P* < .001; adjusted *R*^2^ = .694; Supplemental Table 2).

## Discussion

This study extends prior work on CCR in MS by moving beyond treatment “dose” (ie, duration, frequency, and adherence) to evaluate which specific RehaCom modules contribute to cognitive improvement and which participant characteristics predict responsiveness. Using CogEx data, we examined module progression alongside baseline demographic and cognitive characteristics in progressive MS. Several key findings emerged.

First, progression in attention-focused modules—particularly Attention/Concentration and Divided Attention-2—was significantly associated with improvements in processing speed. Divided Attention-1 also correlated with SDMT change, though no participants reached its final level, limiting interpretation. Sustained Attention was not significantly associated, despite differences between those who did and did not reach its final level, likely reflecting ceiling effects. These findings suggest that attention-based modules are especially relevant for enhancing processing speed, a core deficit in MS.^11^ No effects were seen for memory outcomes, this suggests that training benefits did not generalize to secondary measures, indicating a lack of transfer rather than an absence of baseline impairment. Future studies may need to incorporate memory-specific modules if gains in this domain are to be targeted.

An important consideration when interpreting these findings is the distinction between near-transfer and far-transfer effects in computerized cognitive rehabilitation. The observed associations between progression in the Attention/Concentration module and SDMT outcomes likely reflect near-transfer, as this module emphasizes visual scanning, rapid discrimination, and sustained attentional control—processes closely aligned with SDMT task demands. In this context, improvement may represent training effects on cognitively similar tasks rather than broader generalization to untrained domains. Whether such gains translate into meaningful real-world functional improvements remains uncertain, as ecologically valid outcomes were not assessed in this study.

Importantly, a meaningful proportion of participants achieved SDMT improvements that meet commonly used criteria for individual-level clinical significance, suggesting that observed gains were not solely statistically significant. Future work incorporating functional, patient-reported, or performance-based measures will be necessary to determine the extent of far-transfer associated with RehaCom training.

Second, regression analyses showed that baseline cognitive capacity and training progression jointly influenced outcomes. In this context, baseline cognitive capacity refers to current processing speed performance (indexed by baseline SDMT), whereas cognitive reserve reflects enduring premorbid intellectual capacity (indexed by premorbid IQ). Baseline SDMT and premorbid IQ were strong predictors of outcome, consistent with evidence that both preserved cognitive capacity and cognitive reserve contribute to rehabilitation responsiveness.^12^ Although baseline BVMT-R performance was significantly correlated with SDMT change in bivariate analyses (Table 3), it did not independently predict SDMT outcomes in multivariable regression models once baseline processing speed and other covariates were included. Together, these findings are consistent with the notion of a potential window of opportunity for cognitive rehabilitation, whereby preserved baseline cognitive capacity and reserve may facilitate responsiveness; however, because this study focused on individuals with established cognitive impairment, it was not designed to evaluate preventive or pre-rehabilitation strategies.

Importantly, progression in both the Attention/Concentration and Divided Attention-2 module independently predicted SDMT gains, highlighting intervention content—not only patient profile—as a determinant of responsiveness. This underscores the potential of precision rehabilitation, where modules are tailored to maximize benefit.

Third, combining Attention/Concentration with both Divided Attention modules provided additional insight. The composite score showed stronger bivariate associations with SDMT than the individual modules, though in regression models explained slightly less variance than separate predictors. This likely reflects loss of unique variance once modules were averaged, despite strong intercorrelations. Because CogEx presented modules in a fixed interleaved sequence, the individual contribution of each module and of progression within them cannot be fully disentangled. Future research should test whether delivering these modules sequentially alters the strength or durability of CCR gains.

Fourth, task difficulty varied considerably. Vigilance-2 and Sustained Attention were prone to ceiling effects, while Divided Attention modules appeared overly complex, preventing participants from reaching final levels. Attention/Concentration achieved the best balance, aligning with evidence that adaptive task difficulty promotes engagement, neural plasticity, and transfer of training gains.^13^ It is also possible that variability in the number of difficulty levels across RehaCom modules influenced sensitivity to change, as modules with fewer levels may offer less gradation in difficulty, whereas modules with a broader difficulty range may better capture individual differences in progression.

Fifth, 6-month follow-up showed that baseline SDMT, Attention/Concentration, and Divided Attention-2 remained significant predictors. Female sex and older age were also associated with higher SDMT scores, suggesting demographic contributions to longer-term outcomes. The delayed effect of sex implies that men may have experienced greater decline over time.

Findings align with prior work. Kever et al^14^ and Sandroff et al^3^ reported that fewer failed tests, intact memory, and higher premorbid IQ predicted responsiveness. Taylor et al^4^ identified younger age, relapsing–remitting MS, and milder impairment as favorable, while Ziccardi et al^15^ found that single-domain impairment predicted greater benefit than multi-domain deficits. Similar patterns are evident in aging, where individuals with milder impairment respond better to CCR.^16^ Together, these studies emphasize the importance of baseline cognitive profile and reserve.

Traditionally, CCR research has emphasized dose and overall program adherence, potentially overlooking mechanisms of change.^1,2,17^ By linking module-specific progression—particularly attention-focused tasks—to processing speed, this study highlights the value of intervention content in shaping outcomes. Such insights are vital for optimizing scarce rehabilitation resources and tailoring treatment.

Beyond behavioral measures, additional predictors have been reported. Neuroimaging links responsiveness to cerebellar structure and connectivity,^18-20^ while psychosocial factors, including personality traits, may also play a role.^21^ Fuchs et al^22^ showed that greater gray matter volume, stronger executive function, and conscientiousness predicted larger gains. Prouskas et al^5^ emphasized connectivity between default mode and attention networks, while Nauta et al^23^ found that MEG-based network slowing and hyperconnectivity predicted responsiveness. Fuchs et al^24^ demonstrated that reduced disruption of default mode tracts predicted benefit, and Esbrí et al^25^ showed that preserved processing speed, higher reserve, and greater gray matter integrity enhanced outcomes, with thalamic integrity exerting a direct effect. Together, these findings highlight that preserved baseline functional and structural organization is a key determinate of responsiveness, consistent with the notion of a “window of opportunity” for intervention.

### Limitations

Several limitations warrant consideration. First, the sample was restricted to progressive MS, limiting generalizability to relapsing–remitting disease. Second, as a secondary analysis, the study was not powered for these endpoints and findings should be considered exploratory. Third, ceiling effects in Vigilance-2 and Sustained Attention limited their variability and inclusion in regression models. Finally, although CogEx included a sham CCR arm, this analysis did not, so nonspecific influences such as practice effects cannot be fully excluded.

Although these findings are consistent with the concept of a potential window of opportunity for intervention, the present study was not designed to evaluate preventive or pre-rehabilitation strategies, and prospective studies are needed to determine whether earlier intervention confers additional benefit.

## Conclusion

Cognitive rehabilitation in progressive MS is not uniformly effective: not all participants, and not all modules, contribute equally to cognitive gains. Attention/Concentration emerged as a particularly influential module, while baseline cognitive capacity and cognitive reserve, indexed by baseline SDMT performance and premorbid IQ, shaped responsiveness. These findings support a framework for distinguishing responders from non-responders and tailoring CCR accordingly. Future research should integrate behavioral, neuroimaging, and psychosocial predictors to refine precision rehabilitation strategies in MS.

## Supplemental Material

sj-docx-1-nnr-10.1177_15459683261432556 – Supplemental material for Looking Beyond Dose: Identifying Responders and Non-Responders to RehaCom Computerized Cognitive Rehabilitation in Progressive MS—The CogEx Study Supplemental material, sj-docx-1-nnr-10.1177_15459683261432556 for Looking Beyond Dose: Identifying Responders and Non-Responders to RehaCom Computerized Cognitive Rehabilitation in Progressive MS—The CogEx Study by Cecilia Meza, Roberto S. Hernandez, Silvana L. Costa, Amber Salter, Nancy D. Chiaravalloti, Maria Pia Amato, Giampaolo Brichetto, Jeremy Chataway, Gary Cutter, Ulrik Dalgas, John DeLuca, Rachel Farrell, Peter Feys, Massimo Filippi, Jennifer Freeman, Matilde Inglese, Robert W. Motl, Maria A. Rocca, Brian M. Sandroff and Anthony Feinstein in Neurorehabilitation and Neural Repair
