# Supplementary material for: Looking Beyond Dose: Identifying Responders and Non-Responders to RehaCom Computerized Cognitive Rehabilitation in Progressive MS—The CogEx Study
Source: Neurorehabil Neural Repair. 2026 Apr 25;40(7):575–86. doi: 10.1177/15459683261432556 (PMC13291402; doi:10.1177/15459683261432556)
Supplement: sj-docx-1-nnr-10.1177_15459683261432556 – Supplemental material for Looking Beyond Dose: Identifying Responders and Non-Responders to RehaCom Computerized Cognitive Rehabilitation in Progressive MS—The CogEx Study [file sj-docx-1-nnr-10.1177_15459683261432556.docx]

| **Supplementary Figure 1.** RehaCom Module Tracking Sheet | | | | |
| --- | --- | --- | --- | --- |
| **Participant ID: Examiners Initials:** | | | | |
| Week | Session | Module 1 | Module 2 | Level End |
| 1 | 1 | Attention / Concentration | Sustained Attention |  |
| 1 | 2 | Sustained Attention | Divided Attention 1 |  |
| 2 | 3 | Divided Attention 1 | Divided Attention 2 |  |
| 2 | 4 | Divided Attention 2 | Vigilance 2 |  |
| 3 | 5 | Vigilance 2 | Attention / Concentration |  |
| 3 | 6 | Attention / Concentration | Sustained Attention |  |
| 4 | 7 | Sustained Attention | Divided Attention 1 |  |
| 4 | 8 | Divided Attention 1 | Divided Attention 2 |  |
| 5 | 9 | Divided Attention 2 | Vigilance 2 |  |
| 5 | 10 | Vigilance 2 | Attention / Concentration |  |
| 6 | 11 | Attention / Concentration | Sustained Attention |  |
| 6 | 12 | Sustained Attention | Divided Attention 1 |  |
| 7 | 13 | Divided Attention 1 | Divided Attention 2 |  |
| 7 | 14 | Divided Attention 2 | Vigilance 2 |  |
| 8 | 15 | Vigilance 2 | Attention / Concentration |  |
| 8 | 16 | Attention / Concentration | Sustained Attention |  |
| 9 | 17 | Sustained Attention | Divided Attention 1 |  |
| 9 | 18 | Divided Attention 1 | Divided Attention 2 |  |
| 10 | 19 | Divided Attention 2 | Vigilance 2 |  |
| 10 | 20 | Vigilance 2 | Attention / Concentration |  |
| 11 | 21 | Attention / Concentration | Sustained Attention |  |
| 11 | 22 | Sustained Attention | Divided Attention 1 |  |
| 12 | 23 | Divided Attention 1 | Divided Attention 2 |  |
| 12 | 24 | Divided Attention 2 | Vigilance 2 |  |

| **Supplementary Table 1.** Multivariable regression analysis predicting 6 month SDMT scores using individual RehaCom module progression variables. | | | |
| --- | --- | --- | --- |
| Predictor Variable | β | 95% CI | p-value |
| Age | 0.22 | 0.06, 0.37 | **0.006** |
| Sex |  |  |  |
| Male (ref.) | — | — |  |
| Female | 2.6 | 0.19, 5.0 | **0.034** |
| Baseline SDMT score | 0.69 | 0.49, 0.88 | **<0.001** |
| Max Level % – Attention/Concentration | 0.21 | 0.09, 0.33 | **<0.001** |
| Max Level % – Divided Attention 2 | 0.15 | 0.05, 0.25 | **0.005** |
| Adjusted R² = 0.705 | | | |
| Index 2 = maximum difficulty attained, expressed as a percentage of the module’s maximum.  Stepwise linear regression with AICc-based selection was applied.  Compared with the 12-week model (Table 5A), age and sex emerged as significant predictors at 6 months, while premorbid IQ no longer contributed uniquely. Divided Attention-1, although significantly correlated with SDMT change in bivariate analyses, was not included in regression models and was instead incorporated into the composite attention measure presented in Table 5B. The final model explained 70.5% of the variance in 6-month SDMT scores (adjusted R² = 0.705).  Candidate predictors were identical to those used in the 12-week models; variables not shown were tested but not retained by AICc-based selection. | | | |

| **Supplementary Table 2.** Multivariable regression analysis predicting 6 month SDMT scores using a composite measure of RehaCom attention modules. | | | |
| --- | --- | --- | --- |
| Predictor Variable | β | 95% CI | p-value |
| Age | 0.19 | 0.03, 0.35 | **0.018** |
| Sex |  |  |  |
| Male (ref.) | — | — |  |
| Female | 2.5 | 0.15, 4.9 | **0.037** |
| Baseline SDMT score | 0.76 | 0.57, 0.94 | **<0.001** |
| Max Level % - Attention/Concentration, Divided Attention 1, Divided Attention 2 composite | 0.32 | 0.22, 0.42 | **<0.001** |
| Adjusted R² = 0.694 | | | |
| Composite Index 2 = average percentage of maximum difficulty attained across Attention/Concentration, Divided Attention-1, and Divided Attention-2 modules.  At 6 months, baseline SDMT, age, sex, and the composite attention measure were independent predictors of SDMT outcomes. This differs from the 12-week composite model (Table 5B), where age did not reach significance, indicating that demographic factors contributed more strongly to longer-term outcomes. The final model explained 69.4% of the variance in 6-month SDMT scores (adjusted R² = 0.694).  This model used the same candidate predictor pool as the 12-week composite model; differences reflect timepoint-specific retention rather than changes in model specification. | | | |

| **Supplementary Table 3.** SDMT Change Scores and Proportion Achieving Clinically Meaningful Improvement in the Full Sample and RehaCom Subgroup | | |
| --- | --- | --- |
| **Characteristic** | Total CogEx cohort | CogEx RehaCom subgroup analyzed |
|  | **N = 311***^1^* | **N = 153***^1^* |
| SDMT Change |  |  |
| Mean (SD) | 6 (8) | 6.3 (6.3) |
| Median [Q1, Q3] | 6 [1, 10] | 6.0 [2.0, 10.0] |
| Min, Max | -22, 36 | -9.0, 29.0 |
| Unknown | 27 | 9 |
| SDMT Change (≥8 pts) | 106 (37%) | 57 (40%) |
| Unknown | 27 | 9 |
| *^1^* n (%)  SDMT change scores represent the difference between follow-up (12-week) and baseline performance. Clinically meaningful improvement was defined as an increase of **≥8 points on the SDMT**, consistent with prior CogEx analyses. Percentages are calculated excluding participants with missing SDMT data. | | |
